# 

**Published:** 1866-09

**Authors:** 


### Books and Pamphlets Received.

Manual of Materia Medica and Therapeutics; being an abridgment of the late Dr. Pereira's Elements of Materia Medica, by Frederic John Farre, M. D., Cantab , F. L. S., Fellow of the Royal College of Physicians of London, etc., assisted by Robert Bentley, M. R. C. S., F. L., Honorary Fellow of King's College, London, etc., and by Robert Warington, F. R. S., F. C. S., Chemical Operator to the Society of Apothecaries, and Vice President of the Chemical Society. Edited, with numerous references to the U. S. Pharmacopœia, and many other additions, by Horatio C. Wood, jr., M. D., Professor of Botany, University of Pennsylvania, Auxiliary Faculty of Medicine; Member of the American Philosophical Society; Recording Secretary of the Academy of Natural Sciences; Fellow of the College of Physicians of Philadelphia; Corresponding Member of the New York Lyceum of Natural History, etc., etc., with two hundred and thirty-six wood engravings. Philadelphia: Henry C. Lea.

A Manual of the Principles of Surgery, based on Pathology, for Students. By William Canniff, Licentiate of the Medical Board of Upper Canada; M. D. of the University of New York: M. B. C. S., England; formerly House Surgeon to the Seamen's Hospital, New York; for some time A. A. Surgeon to Her Britannic Majesty's forces; late Professor of General Pathology and the Principles and Practice of Surgery, Univ. Victoria College, C. W.; and for a short time A. A. Surgeon to the U. S. Army. Philadelphia: Lindsay & Blakiston.

A Guide to the Practical Study of Diseases of the Eye; with an outline of their Medical and Operative Treatment. By James Dixon, F. R. C. S., Surgeon to the Royal London Ophthalmic Hospital, Moorfields. From the third London edition. Philadelphia: Lindsay & Blakiston.

Asiatic Cholera; a Treatise on its Origin, Pathology, Treatment, and Cure. By E. Whitney, M. D., and A. B. Whitney, A. M., M. D., late Physician and Surgeon to Diseases of Women in the North-Western Dispensary, Visiting Physician, etc. New York: M. W. Dodd, Publisher, 506 Broadway.

Clinical Lectures by Prof. A. von Graefe, on Amblyopia and Amaurosis, and the Extraction of Cataract. Translated from the German by Hasket Derby, M. D.

The Hunterian Ligation of Arteries to Relieve and to Prevent Destructive Inflammation; by Henry F. Campbell, M. D., Augusta, Ga.

Disinfectants; by E. R. Squibb, M. D., Brooklyn, N. Y.

An Introduction to the Study of the Optical Defects of the Eye, and their Treatment by the Scientific use of Spectacles. By A. M. Rosebrugh, M. D., Toronto.

Epidemic Cholera; its Pathology and Treatment. By A. B. Palmer, M. D., Professor of Pathology, the Practice of Medicine, and of Hygiene, in the University of Michigan, etc.

On Excision of the Superior Maxilla; report of a case with remarks on certain Tumors of this Bone. By Wm. R. Whitehead, M. D., (Univ. of Paris.)

Annual Announcement of the Medical Department of the University of Buffalo, for the Session of 1866-67.

Transactions of the Indiana State Medical Society, at its Sixteenth Annual Session, held at Indianapolis, May 15th, 16th and 12th, 1866.

Seventh Annual Announcement of Miami Medical College, Cincinnati, for the Session of 1866-67.

Transactions of the Vermont Medical Society, for the year 1865.

Prospectus of the Course of Instruction in the Humbolt Medical College, (Saint Louis, Mo.) Winter Session commencing Sept. 17, 1866.

College of Physicians and Surgeons, New York. Medical Department of Columbia College. Annual Announcement. Sixtieth Session, 1866-7.

University of New York—Medical Department—Annual Announcement of Lectures—Session 1866-67.

Annual Announcement of the Faculty of Medicine of the McGill University, Montreal, for the Thirty-third Session, 1865-66.

### Exchange Journals.

The following Journals are regularly received in exchange:

London Lancet—Editors, J. H. Bennett, M. D., T. Wakely, jr., M. R. C. S. E.

Braithwaite's Retrospect of Practical Medicine and Surgery. New York: W. A. Townsend, 434 Broome street.

The Ophthalmic Review, edited by J. Z. Lawrence and Thomas Winslow, London.

American Journal of the Medical Sciences, edited by Isaac Hays, M. D.

Boston Medical and Surgical Journal, edited by Samuel L. Abbott, M. D. and James C. White, M. D.

The American Journal of Insanity, edited by the officers of the New York State Lunatic Asylum.

The New York Medical Journal.

The Cincinnati Lancet and Observer, edited by Edward B. Stevens, M. D. and John A. Murphy, M. D.

The St. Louis Medical and Surgical Journal, edited by M. L. Linton, M. D. and Frank W. White, M. D.

The Medical Record.

The Chicago Medical Journal.

The Chicago Medical Examiner, edited by N. S. Davis, M. D..

The Medical and Surgical Reporter, edited by S. W. Butler, M. D.

The Cincinnati Journal of Medicine, edited by George C. Blackman, M. D., Theophilus Parvin, M. D. and Roberts Bartholow, M. D.

The Richmond Medical Journal, edited by E. S. Gaillard, M. D. and W. S. McChesney, M. D.

The Savannah Journal of Medicine, edited by Juria Harris, M. D., J. B. Read, M. D. and J. G. Thomas, M. D.

The Medical Reporter, edited by J. S. B. Alleyne, M. D. and O. F. Potter, M. D.

The Detreit Review of Medicine and Pharmacy, edited by George T. Andrews, M. D., S. T. Duffield, M. D. and E. W. Jenks, M. D.

Atlanta Medical and Surgical Journal, edited by J. G. Westmoreland, M. D.

The Medical and Surgical Monthly, Memphis, Tenn., edited by Frank A. Ramsay, M. D., D. D. Saunders, M. D., E. Mills Willett, M. D. and William H. White, M. D.

Southern Journal of Medical Sciences, edited by E, D. Turner, M. D., D. Warren Brickell, M. D. and C. Beard, M. D.

The Pacific Medical and Surgical Journal and Press, edited by Henry Gibbons, M. D.

The Galveston Medical Journal, edited by Greenvill Dowell, M. D.

The New Orleans Medical Record, a semi-monthly Journal of the Medical Sciences, edited by Bennett Dowler, M. D. and S. R. Chambers, M, D.

The American Journal of Pharmacy, edited by William Proctor, jr.

Medical News and Library, by Henry C. Lea, Philadelphia.

The Druggist's Circular and Chemical Gazette; The Journal of Materia Medica, by Joseph Bates, M. D. and H. A. Tilden.

The Dental Cosmos, edited by J. H. McQuillen, D. D. S. and George J. Zeigler, M. D.

The Atlantic Monthly, published by Ticknor, Fields & Co., Boston.

Godey's Lady's Book, published by Louis A. Godey, Philadelphia.

The Herald of Health.

Eclectic Medical and Surgical Journal, Philadelphia.

Eclectic Medical Journal, Cincinnati.

American Eclectio Medical Review, New York.

Eclectic Medical Journal, Cincinnati, Ohio.

Philadelphia University Journal.

Dental Register, Cincinnati.

The Nation.

Every Saturday, published by Ticknor, Fields & Co., Boston.

Educational Monthly.

